# Interaction of Immune Cells and Tumor Cells in Gold Nanorod–Gelatin Composite Porous Scaffolds

**DOI:** 10.3390/nano9101367

**Published:** 2019-09-24

**Authors:** Xiuhui Wang, Naoki Kawazoe, Guoping Chen

**Affiliations:** 1 Tissue Regeneration Materials Group, Research Center for Functional Materials, National Institute for Materials Science, Tsukuba, Ibaraki 305-0044, Japan; WANG.Xiuhui@nims.go.jp (X.W.); KAWAZOE.Naoki@nims.go.jp (N.K.); 2 Department of Materials Science and Engineering, Graduate School of Pure and Applied Sciences, University of Tsukuba, Tsukuba, Ibaraki 305-8571, Japan

**Keywords:** gold nanorods, composite scaffolds, gelatin, photothermal ablation, cell interaction, immune responses, cancer therapy

## Abstract

Composite porous scaffolds prepared by immobilization of photothermal nano-agents into porous scaffold have been used for both cancer therapy and tissue regeneration. However, it is not clear how the host immune cells and ablated tumor cells interact and stimulate each other in the composite scaffolds. In this research, a gold nanorod-incorporated gelatin composite scaffold with controlled spherical large pores and well interconnected small pores was fabricated by using ice particulates as a porogen. The composite porous scaffold was used for investigating the interaction between dendritic cells and photothermally ablated breast tumor cells. The composite scaffold demonstrated excellent photothermal property and the temperature change value could be adjusted by irradiation time and laser power density. The composite scaffold showed excellent photothermal ablation ability towards breast tumor cells. The photothermally ablated tumor cells induced activation of dendritic cells when immature dendritic cells were co-cultured in the composite scaffold. Consequently, the gold nanorod–incorporated gelatin composite porous scaffold should provide a useful platform for simultaneous photothermal-immune ablation of breast tumor.

## 1. Introduction

Breast cancer has become one of the most common cancers due to its increasing morbidity and mortality worldwide [1,2,3]. It is still difficult to realize a complete cure, although some new approaches, such as gene therapy, chemotherapy, photothermal therapy and immunotherapy, have been developed [4,5,6,7,8,9]. Among them, photothermal therapy, which produces heat using photo-absorbing agents under near infrared (NIR) laser irradiation to thermal elimination of tumor cells, affords a newly developed cancer therapeutic strategy [10,11,12]. A large variety of photothermal conversion agents including organic nano-agents, such as NIR dye micelles, and inorganic nano-agents, such as graphene oxide, black phosphorous nanoparticles, iron oxide nanoparticles and gold nanoparticles, have been widely reported [13,14,15,16,17,18]. In particular, gold nanorods (AuNRs), as a kind of typical photothermal conversion agent, have raised much attention because of their unique characteristics including facile synthesis, excellent photothermal conversion efficiency and low cytotoxicity [19,20,21]. However, it is still limited to achieve a specific delivery of free nanoparticles into the tumor site in spite of the adoption of modification of nanoparticles with tumor markers to increase their uptake and accumulation in tumors [22,23,24,25]. To address the issue, photothermal scaffolds by immobilization of photothermal nanoparticles into three-dimensional (3D) scaffolds have been developed to constrain all the nanoparticles in the tumor site [26,27]. The composite scaffolds can be repeatedly irradiated by NIR laser to realize repeated heating to ablate tumor cells [28,29]. However, the composite photothermal scaffolds have been mainly used in the primary tumor by implanting them into the tumor site [26,30]. For metastatic tumors, though, it is still difficult to inhibit their growth and to prevent tumor recurrence.

A new generation of photothermal cancer therapy should not only be able to ablate the primary tumor but also to activate the immune system for attacking metastatic tumor cells as a trigger of cancer immunotherapy [31,32,33]. Previous studies have reported that dead tumor cells including necrotic and apoptotic tumor cells have some effects on the activation of the immune system [34,35]. Necrotic tumor cells can release immune-stimulatory proteins, such as calreticulin (CRT), heat shock protein (HSP) and tumor-specific antigens [36]. Apoptotic tumor cells involved with “heat stress” enhance the membrane HSPs expression [32,37,38]. These signals can activate the immune system to trigger tumor-specific immunity [36,39]. It has been reported that dendritic cells (DCs), as a kind of antigen-presenting cell (APCs), can be effectively activated and become mature by dead tumor cells that are photothermally ablated by free photothermal nanoparticles [40,41,42,43,44]. Subsequently, the mature DCs can recruit and induce activation of tumor-specific T lymphocytes, which further trigger anti-tumor immunotherapy [45,46]. However, it is not clear how immune cells interact with and are activated by photothermally ablated tumor cells in the 3D composite scaffold.

**Scheme 1:**
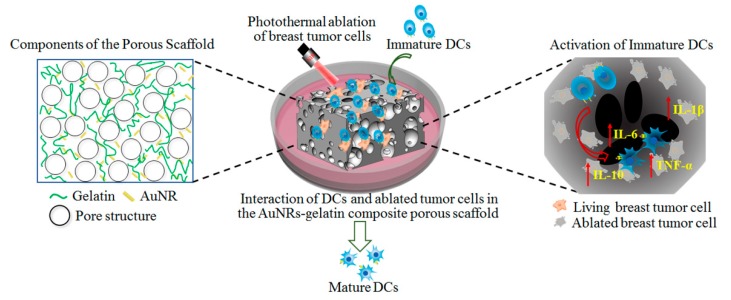
Illustration of the interaction of dendritic cells (DCs) and photothermally ablated tumor cells in gold nanorod-gelatin composite porous scaffolds.

In this work, an AuNRs-incorporated gelatin composite porous scaffold with well controlled pore structures was constructed by using ice particulates as a porogen material. The size of spherical large pores was precisely controlled by optimizing the diameter of ice particulates using sieves with appropriate mesh sizes. The tunable pore size affects cell adhesion, migration, cell-cell interaction and communication. The good interconnectivity of pore structure promotes efficient cell seeding, cellular penetration, nutrition supply and metabolite exchange. The method can be easily scaled up to prepare big scaffolds and a large number of scaffolds for future applications. As shown in Scheme 1, the photothermal conversion property of the AuNRs–gelatin composite porous scaffold and its photothermal ablation efficiency towards breast tumor cells were investigated by NIR laser irradiation. Moreover, the effect of photothermally ablated tumor cells on the activation of DCs in the AuNRs–gelatin composite scaffold was evaluated by co-culturing the immature DCs with ablated tumor cells in the composite scaffold.

## 2. Materials and Methods

### 2.1. Fabrication and Characterization of AuNRs–Gelatin Composite Porous Scaffold

AuNRs were synthesized according to the protocol of a previous study [47]. At first, gold seeds were pre-synthesized through chemical reduction of HAuCl_4_ using NaBH_4_ as a reductant. Subsequently, the gold seeds solution was dropped into a growth solution containing CTAB, HAuCl_4_, HCl, AgNO_3_ and ascorbic acid under gentle stirring. After incubating for 12 h, the solution was centrifuged to obtain AuNRs. Finally, the AuNRs were dispersed in gelatin aqueous solution under stirring to coat gelatin molecules on AuNRs.

The gelatin-coated AuNRs were hybridized with gelatin porous scaffold to prepare AuNRs-gelatin composite porous scaffolds as previously reported [27]. In brief, ice particles were formed via spraying pure water into liquid nitrogen and their size was optimized by sieving them through two kinds of mesh sieves having different mesh sizes (diameter of mesh sizes: 425 and 500 μm). The gelatin-coated AuNRs solution was then dropped into 8 (*w/v*) % gelatin aqueous solution which was prepared by dissolving gelatin powders in 70% acetic acid solution. The suspension solution of AuNRs-gelatin was sonicated to make homogeneous dispersion of AuNRs in the gelatin solution, and the concentration of AuNRs was adjusted at 2.0 mM. Finally, the ice particles and AuNRs-gelatin suspension solution with the ratio of 7:3 (*w/v*) were mixed and transferred in the silicone mold under −4 °C. The construct was frozen in at −20 °C for 12 h and then −80 °C for 4 h, freeze-dried with a freeze-dryer (FDU-2200, Tokyo, Japan), followed by cross-linking to obtain the AuNRs-incorporated gelatin composite porous scaffold.

AuNRs morphology was imaged by a transmission electron microscope (TEM, JEM-2100F, JEOL Ltd., Tsukuba, Japan) and their size was analyzed with ImageJ software. The hydrodynamic size and polydispersity index of gelatin-coated gold nanorods (AuNRs) dispersed in water were measured with a particle size analyzer (ELSZ-2000, Otsuka Electronics Co., Ltd., Osaka, Japan). The gold concentration was analyzed with an inductively coupled plasma-optical emission spectroscopy (ICP-OES, SII Nano Technology Inc., Chiba, Japan). Pore structure of the AuNRs-gelatin composite scaffold was observed using a scanning electron microscope (SEM: SU8220, Hitachi, Japan). Photothermal property of the composite scaffold was evaluated under 805 nm NIR laser irradiation. For the NIR laser irradiation experiment, the hydrated AuNRs-gelatin composite scaffold was put in a culture dish and placed above the NIR laser. Then, the light-induced temperature change of the composite scaffold by laser irradiation at various laser power densities (1.2, 1.4 and 1.6 W cm^−2^) was monitored by an infrared thermal imaging camera (FLIR E5, As one Corp., Tokyo, Japan). The real-time temperature of the scaffold’s center part was displayed on the FLIR monitor. Triplicate samples were used for each measurement (*n* = 3).

### 2.2. Photothermal Ablation Ability of Tumor Cells Cultured in AuNRs–Gelatin Composite Scaffold

The AuNRs-gelatin composite scaffold cubes (5.0 mm × 3.0 mm × 1.0 mm) were sterilized using 70% ethanol followed with thrice washing with sterile PBS. Mouse breast tumor cells (4T1-Luc) were incubated in a tissue culture flask with RPMI1640 serum medium at 37 °C and 5% CO_2_. Upon reaching a sub-confluent state in a flask, the cells were sub-cultured to get enough cells. The sub-cultured 4T1-Luc cells were collected at the concentration of 6.67 × 10^6^ cells/mL. A 15 µL cell suspension was dropped into each side of the scaffold cube (30 µL/both sides, 2 × 10^5^ cells/scaffold). After that, the seeded cells in the composite porous scaffold were cultured in the 24-well culture plate.

After being cultured for 24 h, the 4T1-Luc/scaffold construct was taken out from each well of the culture plate and the excess medium surrounding the constructs was carefully removed by using sterilized Kimwipes paper. Subsequently, the 4T1-Luc/scaffold constructs were transferred into a 96-well culture plate and irradiated under an NIR laser (805 nm, 1.6 W cm^−2^) for 3 min. Before and after laser irradiation, live and dead cells in the constructs were stained with a calcein-AM/PI double staining kit and photographed under an all-in-one fluorescence microscope (BZ-X710, Keyence Corp., Osaka, Japan). Moreover, after being irradiated (805 nm, 1.6 W cm^−2^) for 0, 1, 2 and 3 min, cell viability in the constructs was quantitatively evaluated with a WST-1 assay. Triplicate samples were determined for every experiment (*n* = 3).

### 2.3. Co-Culture of Immature DCs and Photothermally Ablated Tumor Cells in AuNRs–Gelatin Composite Scaffold

The 4T1-Luc cells (2 × 10^5^ cells per scaffold) were cultured in the composite scaffold cubes for 2 days. Subsequently, the 4T1-Luc/scaffold constructs were removed from the culture medium and treated as above-mentioned for laser irradiation (805 nm, 1.6 W cm^−2^) for 3 min to thermally eradicate all the tumor cells. In addition, mouse bone marrow-derived immature DCs were incubated in the tissue culture flask with MEM-α serum medium supplemented with GM-CSF. After reaching confluence, the DCs were collected with a concentration of 6.67 × 10^6^ cells/mL. Subsequently, 15 µL DCs suspension solution was dropped in the photothermally ablated 4T1-Luc/scaffold constructs to seed DCs in the constructs (1 × 10^5^ cells/scaffold). The DCs were co-cultured with the ablated 4T1-Luc in the composite scaffold cubes for 36 h. As a control group, DCs (1 × 10^5^ cells/scaffold) were seeded in the living 4T1-Luc/scaffold constructs that were cultured as above-mentioned for 36 h without laser irradiation. As another two control groups, DCs (1 × 10^5^ cells/scaffold) were seeded in the composite scaffold without 4T1-Luc cells and cultured with or without 1 μg/mL lipopolysaccharide (LPS) stimulation for 36 h. Finally, supernatants of all the four types of samples (one experiment sample and three controls) were collected to detect the secretion level of interleukin 6, 10, 1β (IL-6, IL-10, IL-1β) and tumor necrosis factor alpha (TNF-α) using ELISA kit (R&D systems, Biotech, Minneapolis, McKinley Place, MN, USA). Triplicate samples were determined for every experiment (*n* = 3).

### 2.4. Statistical Analysis

Statistical analysis comprising of significant difference was implemented by one-way analysis of variance (ANOVA). The *p* value of 0.05 was regarded as a statistically significant difference. The data were categorized based on *p* value and indicated with *: *p* < 0.05, **: *p* < 0.01 and ***: *p* < 0.001.

## 3. Results and Discussion

### 3.1. Characterization of AuNRs and AuNRs–Gelatin Composite Porous Scaffold

**Figure 1:**
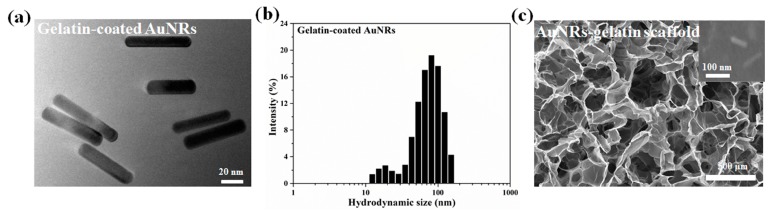
Transmission electron microscope (TEM) image of gelatin-coated gold nanorods (AuNRs) (**a**); hydrodynamic size distribution of gelatin-coated gold nanorods (AuNRs) (**b**); scanning electron microscope (SEM) image of AuNRs–gelatin composite porous scaffold (**c**), insert in c is the magnified image of the pore surface showing the embedding of AuNRs in gelatin matrices.

As shown in Figure 1a, the gelatin-coated gold nanoparticles had a rod-like structure with a nano-scaled dimension of 64.3 ± 3.4 nm × 14.1 ± 2.3 nm. The gelatin-coated AuNRs were uniformly distributed without aggregation. Moreover, the dynamic light scattering (DLS) results (Figure 1b) showed that the average hydrodynamic size of gelatin-coated AuNRs dispersed in water was 69.8 ± 7.5 nm and the polydispersity index (PDI) was 0.295 ± 0.020, which indicated the gelatin-coated AuNRs had a narrow size distribution and good monodispersity in water. The gelatin-coated AuNRs were introduced in gelatin sponge to prepare AuNRs-gelatin composite porous scaffold. SEM observation (Figure 1c) showed the porous structure of the composite scaffold. The composite scaffold showed homogenously distributed spherical large pores with good interconnectivity. Magnified images (insert in Figure 1c) displayed that AuNRs were embedded into the gelatin matrices and homogeneously distributed on the pore walls of the composite scaffold without aggregation.

### 3.2. Photothermal Property of AuNRs–Gelatin Composite Scaffold

**Figure 2:**
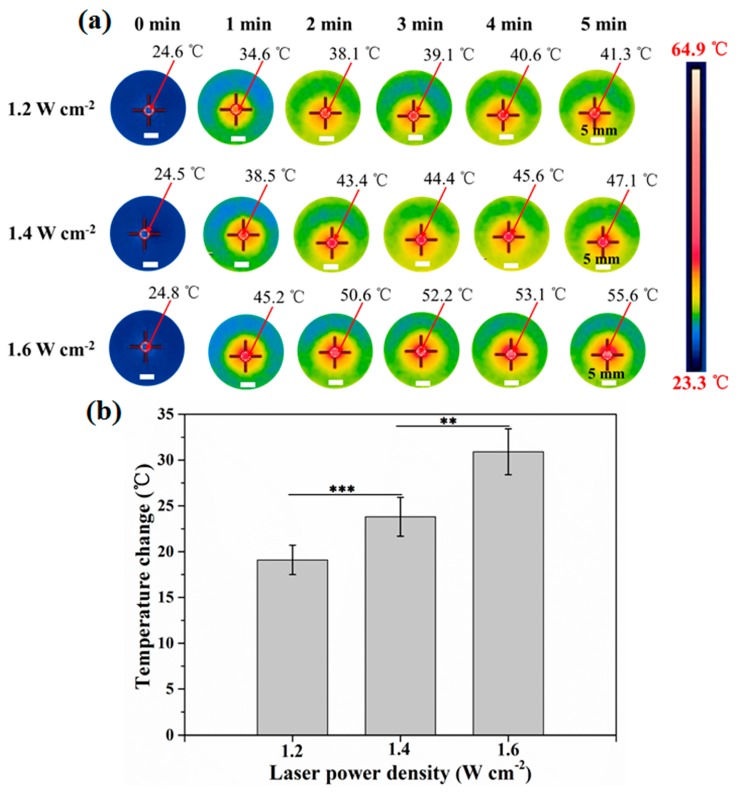
Real-time infrared thermal images of AuNRs–gelatin composite scaffold under 805 nm laser irradiation at various power densities of 1.2, 1.4 and 1.6 W cm^−2^ for 0–5 min (**a**); and temperature change of the AuNRs–gelatin composite scaffold after being irradiated at various laser power densities for 5 min (**b**). The data represent the means ± S.D. (*n* = 3). Significant difference: ** *p* < 0.01; *** *p* < 0.001.

Heating effect of AuNRs in the composite scaffold was confirmed with NIR laser irradiation. The real-time infrared thermal images (Figure 2a) showed that the temperature of the AuNRs-gelatin composite scaffold increased under laser irradiation. The temperature of the composite scaffold increased with the increase of irradiation time. When the composite scaffold cubes were irradiated for the same time (5 min) but at different power density of laser, the temperature change increased with enhancing laser power density (Figure 2b). The temperature change of the composite scaffold cubes was 19.1 ± 1.6 °C, 23.8 ± 2.1 °C, 30.9 ± 2.5 °C, respectively, when the laser power density was controlled at 1.2, 1.4 and 1.6 W cm^−2^. The results indicated that the light-induced temperature increase of the composite scaffold could be adjusted via altering the power density of laser and irradiation time. Higher laser power intensity and longer irradiation time resulted in bigger temperature changes.

The AuNRs–gelatin composite scaffold showed a good photothermal property, which should be the result of the photothermal characteristic of the embedded AuNRs. Previous studies have reported that gold nanoparticles with rod-like morphology have an obvious surface plasmon resonance (SPR) absorption peak within NIR light region and can effectively convert NIR light into heat under 805 nm laser irradiation [40]. The AuNRs in the pore walls of the composite scaffold kept the photothermal conversion capacity after hybridization with gelatin porous scaffold. These results suggested that the AuNRs-gelatin composite scaffold could be used for photothermal ablation of tumor cells.

### 3.3. Photothermal Ablation of Tumor Cells Cultured in AuNRs–Gelatin Composite Porous Scaffold

**Figure 3:**
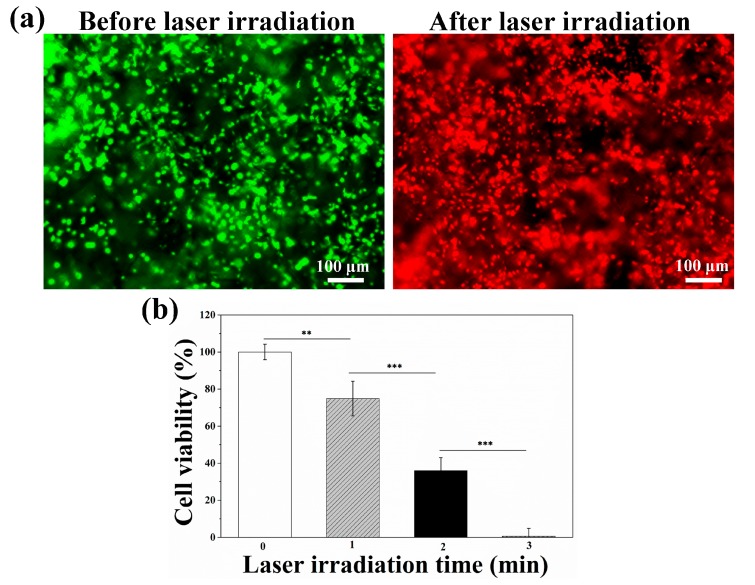
Live/dead staining images of 4T1-Luc cells cultured in the AuNRs–gelatin composite porous scaffolds before and after laser irradiation (805 nm, 1.6 W cm^−2^) for 3 min (**a**). Green color represents live cells while red color represents dead cells. Viability of 4T1-Luc cells cultured in the AuNRs-gelatin composite porous scaffold after laser irradiation for different time (**b**). The data represent the means ± S.D. (*n* = 3). Significant difference: ** *p* < 0.01; *** *p* < 0.001.

After the 4T1-Luc cells were cultured in the composite scaffold cubes for 24 h, the photothermal ablation capability of the composite scaffold was evaluated by NIR laser irradiation. Live/dead staining images (Figure 3a) displayed that almost all the 4T1-Luc cells were alive in the composite scaffold before laser irradiation. After being irradiated for 3 min, almost all the tumor cells were dead. Quantitative analysis of cell viability suggested that cell viability of 4T1-Luc cells cultured in the composite scaffold obviously decreased with prolonging of the NIR laser irradiation time (Figure 3b). After being irradiated for 3 min, cell viability of 4T1-Luc cells decreased to 0%. All the results suggested that the AuNRs-gelatin composite porous scaffold could effectively ablate breast tumor cells under NIR laser irradiation and that the tumor cells’ ablation capacity was dependent on the irradiation time, which should be the result of the photothermal property of the AuNRs embedded in the porous scaffold.

### 3.4. Interaction and Activation of Immature DCs with Photopthermally Ablated Tumor Cells in AuNRs–Gelatin Composite Porous Scaffold

**Figure 4:**
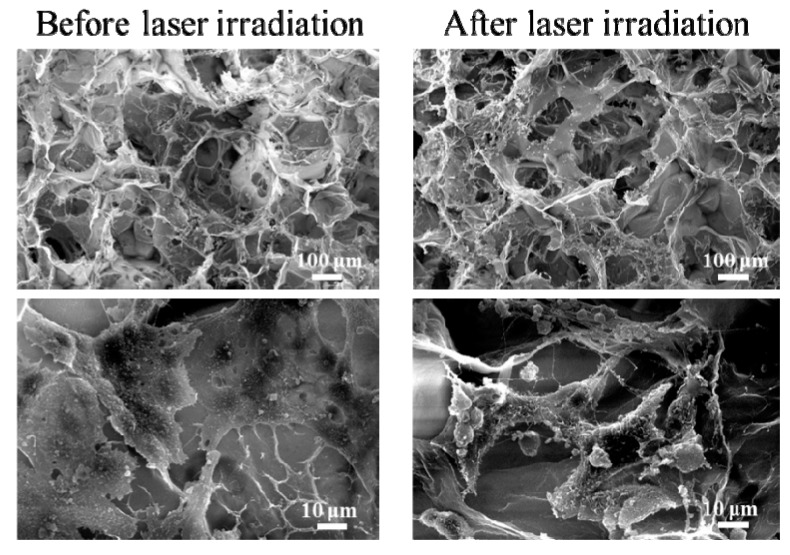
Distribution and morphology of breast tumor cells in the AuNRs–gelatin composite scaffold before and after laser irradiation (805 nm, 1.6 W cm^−2^) for 3 min.

SEM images (Figure 4) showed that, before laser irradiation, the breast tumor cells were homogenously distributed throughout the AuNRs–gelatin composite scaffold. Magnified images showed the tumor cells attached and spread well on the walls of pores in the composite scaffolds. After NIR laser irradiation, the ablated tumor cells still remained on the pore walls of the composite scaffolds.

**Figure 5:**
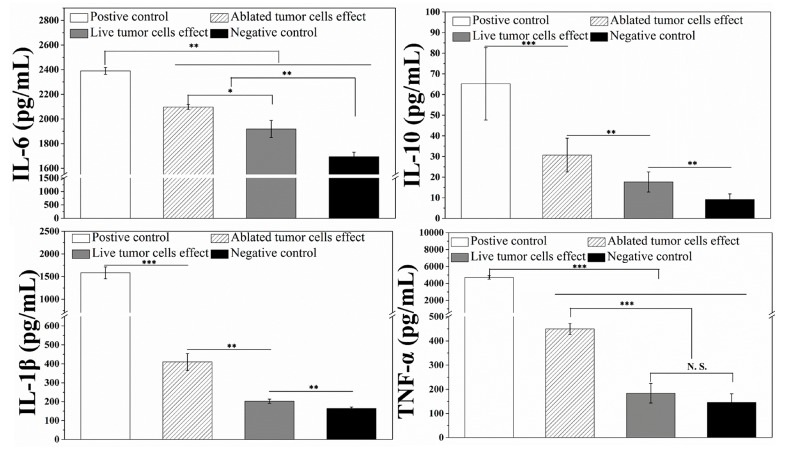
Secretion level of IL-6, IL-10, IL-1β and TNF-α by dendritic cells (DCs) after being co-cultured with living 4T1-Luc cells and photothermally ablated 4T1-Luc cells in the AuNRs-gelatin composite porous scaffold. Positive and negative controls are DCs cultured in the composite scaffolds with and without LPS, respectively. The data represent the means ± S.D. (*n* = 3). No significant difference: N.S.; significant difference: * *p* < 0.05; ** *p* < 0.01; *** *p* < 0.001.

Activation of DCs induced by photothermally ablated tumor cells in the composite scaffold was evaluated by detecting the secretion level of multiple cytokines from DCs. Immature DCs were co-cultured with living 4T1-Luc cells and photothermally ablated 4T1-Luc cells in the composite scaffold. Immature DCs cultured in the composite scaffold without 4T1-Luc cells were used as a positive control (supplement of LPS in culture medium) and a negative control (no LPS in culture medium). The cytokines were detected with ELISA Kit. As shown in Figure 5, the positive control showed the highest secretion, while the negative control showed the lowest secretion of IL-6, IL-10, IL-1β and TNF-α. Compared with the co-culture of DCs with living 4T1-Luc cells, co-culture of DCs with photothermally ablated 4T1-Luc cells in the composite scaffold showed significantly higher secretion of IL-6, IL-10, IL-1β and TNF-α. The results indicate that the photothermally ablated 4T1-Luc cells in the composite scaffolds could interact with immature DCs and activate them to secret multiple cytokines.

Dendritic cells (DCs), especially mature DCs, play a vital role in the activation of primary immunity [48]. The high secretion of multiple cytokines is a key factor in the activation of mature DCs [41]. Mature DCs can present tumor-specific antigen to T cells and further activate T cells to achieve tumor-specific immunotherapy [49,50,51]. Previous study has shown that secretion of IL-6 and IL-1β, which are typical markers of humoral immunity, can be supported by both soluble factors and cell–cell interaction. Secretion of TNF-α, which is a typical marker of cellular immunity, can only be promoted by cell–cell interaction [40,52,53]. SEM observation showed the photothermally ablated 4T1-Luc cells remained in the composite scaffolds. They could directly interact with the co-cultured DCs both through direct cell-cell interaction and through the soluble factors released from the dead 4T1-Luc cells. The combination of photothermal ablation and activation of immune cells by the photothermal ablated breast tumor cells should provide a prospective strategy to eradicate tumor cells.

## 4. Conclusions

A composite porous scaffold of gold nanorods and gelatin with controlled pore size and good interconnectivity was prepared. The size of large micropores in the composite scaffolds was well controlled by optimizing the diameter of ice particulates. Gold nanorods were homogeneously distributed in the composite scaffold. The gold nanorod-gelatin composite porous scaffold showed excellent photothermal performance and good photothermal ablation ability towards breast tumor cells. Moreover, the photothermally ablated tumor cells induced dendritic cells activation by co-culturing immature dendritic cells with ablated tumor cells in the composite scaffold, which should be useful to trigger the immune system to prevent tumor metastasis and recurrence. Consequently, the AuNRs–gelatin composite porous scaffold will provide a potential for effective photothermal-immuno tumor ablation for cancer therapy.
